# Determinants of empirical antipseudomonal antibiotic prescription for adults with pneumonia in the emergency department

**DOI:** 10.1186/s12890-020-1115-0

**Published:** 2020-04-03

**Authors:** Nuria Angrill, Miguel Gallego, Juli Font, Jordi Vallés, Anisi Morón, Eduard Monsó, Jordi Rello

**Affiliations:** 1 Department of Respiratory Medicine, Parc Taulí Hospital Universitari, Institut d’Investigació i Innovació Parc Taulí I3PT, Universitat Autònoma de Barcelona, Sabadell, Spain; 2 CIBER de Enfermedades Respiratorias (CIBERES), Instituto de Salud Carlos III, Madrid, Spain; 3 Emergency Department, Parc Taulí Hospital Universitari, Institut d’Investigació i Innovació Parc Taulí I3PT, Universitat Autònoma de Barcelona, Sabadell, Spain; 4 Critical Care Center, Parc Taulí Hospital Universitari, Institut d’Investigació i Innovació Parc Taulí I3PT, Universitat Autònoma de Barcelona, Sabadell, Spain; 5 Department of Pharmacy, Parc Taulí Hospital Universitari, Institut d’Investigació i Innovació Parc Taulí I3PT, Universitat Autònoma de Barcelona, Sabadell, Spain; 6 Vall d’Hebron Institute of Research (VHIR), Barcelona, Spain; 7 Research Department, CHU Nîmes, Université Montpellier-Nîmes, Nîmes, France

**Keywords:** Pneumonia, *P aeruginosa*, Antipseudomonal antibiotics, Levofloxacin, Guidelines

## Abstract

**Background:**

Antipseudomonal antibiotics should be restricted to patients at risk of *Pseudomonas aeruginosa* infection. However, the indications in different guidelines on community-acquired pneumonia (CAP) are discordant. Our objectives were to assess the prevalence of antipseudomonal antibiotic prescriptions and to identify determinants of empirical antibiotic choices in the emergency department.

**Methods:**

Observational, retrospective, one-year cohort study in hospitalized adults with pneumonia. Antibiotic choices and clinical and demographic data were recorded on a standardized form. Antibiotics with antipseudomonal activity were classified into two groups: a) β-lactam antipseudomonals (β-APS), including carbapenems, piperacillin / tazobactam or cefepime (in monotherapy or combination) and b) monotherapy with antipseudomonal quinolones.

**Results:**

Data were recorded from 549 adults with pneumonia, with *Pseudomonas aeruginosa* being isolated in only nine (1.6%). Most (85%) prescriptions were compliant with SEPAR guidelines and 207 (37%) patients received antibiotics with antipseudomonal activity (14% β-APS and 23% levofloxacin). The use of β-APS was independently associated with ICU admission (OR 8.16 95% CI 3.69–18.06) and prior hospitalization (OR 6.76 95% CI 3.02–15.15), while levofloxacin was associated with negative pneumococcal urine antigen tests (OR 3.41 95% CI 1.70–6.85) but negatively associated with ICU admission (OR 0.26 95% CI 0.08–0.86). None of these factors were associated with *P aeruginosa* episodes. In univariate analysis, prior *P aeruginosa* infection/colonization (2/9 vs 6/372, *p* = 0.013), severe COPD (3/9 vs 26/372, *p* = 0.024), multilobar involvement (7/9 vs 119/372, *p* = 0.007) and prior antibiotic (6/9 vs 109/372, *p* = 0.025) were significantly associated with *P aeruginosa* episodes.

**Conclusions:**

Antipseudomonal prescriptions were common, in spite of the very low incidence of *Pseudomonas aeruginosa*. The rationale for prescription was influenced by both severity-of-illness and pneumococcal urine antigen test (levofloxacin) and prior hospitalization and ICU admission (β-APS). However, these factors were not associated with *P aeruginosa* episodes. Only prior *P aeruginosa* infection/colonization and severe COPD seem to be reliable indicators in clinical practice.

## Background

The clinical practice guidelines recommend that antimicrobial treatment for patients with community-pneumonia (CAP) should be prescribed empirically after evaluation of severity at presentation and pre-existing comorbid disease, and should be stratified based on prognostic risk scores [1–4]. However, the recommendations are inconsistent and compliance is low; the choice of antibiotic treatment at the bedside is a complex process influenced by several factors that have not yet been definitively characterized. Factors proposed in the literature include previous hospitalization, previous antibiotic exposure and underlying diseases, but there are others that are dependent on hospital organization, local patterns of resistance, background speciality and cost-effectiveness [5–7].

Compliance of antibiotic treatment with the guidelines has been associated with improved outcomes, but mortality rates rise in patients at risk of multi-resistant microorganisms such as *P aeruginosa* [8, 9]. However, antipseudomonal antibiotics are considered as broad spectrum and, due to their potential for causing collateral damage [10, 11], their administration should be restricted.

Pneumonia due to *P aeruginosa* occurs in several distinct syndromes such as community-acquired pneumonia (CAP) and bacteremia in neutropenic hosts, and in intubated patients [12] In CAP, coverage of *P aeruginosa* is controversial due to the different rates of prevalence reported in the literature [13–18]. Factors such as structural lung diseases (especially bronchiectasis), repeated exacerbations of severe chronic obstructive pulmonary disease (COPD), chronic oral steroid administration, alcoholism and frequent (> 4 courses per year) or recent antibiotic therapy have been associated with *P aeruginosa* isolates [1, 2, 4, 19]. However, a large retrospective study reported that a significant proportion of patients with CAP due to *P aeruginosa* did not present any of these conditions [17]. Although not routinely included in most CAP guidelines, a certain degree of immunocompromise is frequent in patients with community-acquired pneumonia, with neutropenia being a common comorbid condition [20].

The aim of this study was to assess the prevalence of the use of antibiotics with antipseudomonal activity and the determinants of empirical choice, in patients attended for pneumonia in the emergency department. We hypothesizied was that antipseudomonal antibiotics might be prescribed more frequently than needed, and that determinants of prescription differ between quinolones and other choices.

## Methods

Retrospective, observational cohort study of all consecutive patients attended in the emergency department (ED) at Parc Tauli Hospital (682 beds and a reference population of 391,460 inhabitants), between September 2010 and September 2011. The protocol was approved by the Sabadell Hospital Ethics Committee (2014/538).

### Study population

All patients ≥18 were evaluated for study inclusion. Patients were identified by primary ICD-9 codes for pneumonia (480.0–483.99, 485–487) and by respiratory failure or sepsis, and had to meet the study definition of pneumonia prior to inclusion. Hospital admission was considered when the patient remained in the ED more than 24 h before discharge or was transferred to the ward or the ICU. The following cases were excluded: hospital-acquired pneumonia (diagnosed more than 48 h after hospital admission), witnessed aspiration pneumonia, solid organ or bone marrow transplantation, and alternative diagnoses (tuberculosis, cardiac failure or organizing pneumonia).

#### Definitions

*Pneumonia* was defined as the presence of a new alveolar opacity on chest radiography plus one or more of the following: fever (temperature > 38 °C) or hypothermia (temperature < 35 °C), cough with or without sputum production, pleuritic chest pain and altered breath sounds on auscultation.

*Chronic obstructive pulmonary disease (COPD)* was defined as a postbronchodilator FEV1/FVC ratio below 0.7 in accordance with the Global Initiative for Chronic Obstructive Lung Disease (GOLD) criteria in a patient with a smoking habit of more than 10 pack-years [21].

*Immunocompromised group (ICP)* included human immunodeficiency virus infection (HIV), neoplastic disease treated with chemotherapy or radiation therapy in the previous 3 months, hematological malignancy, anesplenia, and immunosuppressive therapy including chronic corticosteroid therapy (> 8 mg/d methylprednisolone or equivalent for more than 30 days) and non-steroid immunosuppressive therapy.

*Health Care-Associated Pneumonia (HCAP)* was defined according to the criteria reported by the 2005 IDSA/ATS Guidelines [22]. Episodes that did not meet the criteria for HCAP or ICP were classified as CAP [23].

#### Data collection

The following parameters were recorded in the ED: age, sex, smoking status, alcohol habits, and drug consumption, comorbidities, antibiotic treatment in the three months prior to ED visit, clinical symptoms, clinical signs, arterial blood gas measurements, chest radiograph findings, laboratory parameters, diagnostic procedures, and empiric antibiotic therapy. Previous functional status was evaluated by Barthel scale obtained through hospital databases and the *Shared Medical Record of Catalonia (*the *CatSalut program*)*,* and the Charlson index was calculated based on data stored in the hospital database [24]. All patients were classified according to the pneumonia severity index (PSI). Intensive care unit (ICU) admission, patient treatment restrictions (do-not-resuscitate orders) and mortality were also recorded.

#### Microbiological evaluation

The hospital protocol included the performance of two blood cultures, respiratory secretions (when feasible) and nasopharyngeal swabs (in suspected cases of *Influenza virus* in epidemic periods). Likewise, pleural fluid was analyzed in case of pleural effusion. Samples were obtained for bacterial culture before starting antibiotic therapy in the ED. Likewise, urine samples for *S pneumoniae* and *Legionella pneumophila* antigen detection were routinely obtained before antibiotic therapy unless urgent antibiotic therapy was mandatory, and tested using the Binax NOW immunochromatography method (Alere BinaxNOW, *Streptococcus pneumoniae* Antigen Card; Alere Inc., Waltham, MA). We considered the presence of positive urine antigens as evidence of a bacterial infection. Serological tests were not used routinely to detect atypical organisms. In patients in whom microbiological results were negative, or who did not undergo microbiological tests, the etiology was considered unknown. Bronchoscopic samples such as tracheobronchial aspirate or bronchoalveolar lavage from the lower airways were obtained in intubated patients.

#### Antimicrobial therapy

Information was obtained on empirical antibiotic therapy given within the first 24 h of admission. The antibiotic regimen chosen by the attending physician was initiated in the ED in accordance with the main national reference guidelines [4]. https://www.archbronconeumol.org/es-linkresolver-normativas-el-diagnostico-el-tratamiento-13074594

*Antipseudomonal β-lactam* (β-APS) was considered as any regimen that included an intravenous antipseudomonal antibiotic: carbapenems (meropenem, imipenem), antipseudomonal cephalosporin (ceftazidime, cefepime) or β-Lactam/ β-lactamase inhibitor (piperacillin–tazobactam) in monotherapy or in combination.

*Antipseudomonal quinolone* was defined when levofloxacin was administered in monotherapy.

*Prior antibiotic therapy* was defined as the exposure to antibiotics above 48 h during the 3 months prior to hospitalization.

Prior *P. aeruginosa* infection/colonization was defined as confirmed infection/colonization within the year prior to hospitalization, available from patients’ records.

#### Statistical analysis

The SPSS statistical package version 21.0 (SPSS; Chicago, Illinois) was used for the statistical analysis. Results for categorical variables were expressed as absolute and relative frequencies, and continuous variables were expressed as mean values and standard deviation (SD). Differences in demographic and clinical characteristics between groups were assessed using the Chi-squared test and Fisher’s exact test for categorical variables. Means were compared using ANOVA or non-parametric tests when distribution was non-normal. Variables that might have influenced the treatment decision were selected for analysis: prior antibiotic exposure, prior hospitalization, nursing home residence, comorbidities such as COPD, bronchiectasis, diabetes mellitus, chronic liver disease, chronic heart failure, and criteria of immunosuppression. Likewise, episode severity, measured by the need for ICU admission, radiological extent and urine antigen detection of *S. pneumoniae* were also considered.

Multivariate analysis using stepwise logistic regression was performed with prescription of empirical β-APS as a dependent variable. Variables showing a univariate association (*p* < 0.05) were included in the model as covariates. The same analysis was run for levofloxacin as dependent variable. Hosmer–Lemeshow goodness-of-fit test was used for model calibration.

Results were expressed as crude and adjusted odds ratios (OR) with 95% confidence intervals (95% CI). The level of significance was set at 0.05 (two-tailed).

## Results

### Cohort description

A total of 704 consecutive episodes of pneumonia in adults (669 patients) were recorded during the study period, 549 of whom required hospitalization. Pneumonia was classified as CAP in 295/549 (53.7%), as ICP in 125 (22.8%) and as HCAP in 129 (23.5%). The main demographic and clinical characteristics of hospitalized patients are shown in Table 1. The mean age of the population was 71.2 (SD 16.4) years; 64.9% were men, mean Charlson comorbidity index was 2.5 (SD 2.3) and mean Barthel scale 85.6 (SD 23). HCAP group had worse functional status and more treatment restrictions than the other groups, while the Charlson index and 30-day mortality were highest in the ICP group.

**Table 1:** Epidemiological and clinical characteristics of hospitalized patients

Variable	Total *n* = 549	CAP *n* = 295	ICP *n* = 125	HCAP *n* = 129
Age, mean ± SD	71.2 ± 16.4	67.9 ± 17.4	68.8 ± 15	81.1 ± 10.6
Gender (male)	356 (64.8)	181 (61.4)	93 (74.4)	82 (63.6)
Current/former smoker	328 (59.7)	173 (59.6)	87 (69.6)	68 (52.7)
Charlson index, mean ± SD	2.5 ± 2.3	1.6 ± 1.7	4.5 ± 2.6	2.4 ± 1.8
Barthel scale, mean ± SD	85.5 ± 23	91.4 ± 18.1	87.5 ± 18.2	70.5 ± 29.5
Diabetes mellitus	164 (29.9)	83 (28.1)	33 (26.4)	48 (37.2)
COPD severity				
GOLD I-III	95(17.3)	56 (19.0)	17 (15.6)	22 (17.1)
GOLD IV	38(7.1)	17 (5.8)	7 (5–6)	15 (11.6)
Bronchiectasis	24 (4.4)	11 (3.7)	6 (4.8)	7 (5.4)
Chronic heart disease	107 (19.5)	49 (16.6)	19 (15.2)	39 (30.2)
Dementia	86 (15.7)	33 (11.2)	13 (10.4)	40 (31)
Cerebrovascular disease	77 (14)	40 (13.6)	9 (7.2)	28 (21.7)
Chronic renal failure	81 (14.8)	34 (11.5)	17 (13.6)	30 (23.3)
Chronic liver disease	44 (8)	13 (4.4)	23 (18.4)	8 (6.2)
PSI risk class				
I-III	181 (33.0)	141 (47.8)	22 (17.6)	18 (14.0)
IV	212 (38.6)	103 (34.9)	49 (39.2)	60 (46.5)
V	156 (28.4)	51 (17.3)	54 (43.2)	51 (39.5)
ICU admission	59 (10.7)	40 (13.6)	16 (12.8)	3 (2.3)
30-day- mortality	52 (9.5)	15 (5.1)	22 (17.6)	15 (11.6)
Do-not-resuscitate orders	86 (15.7)	24 (8.1)	27 (21.6)	35 (27.1)
Prior antibiotic therapy	167 (30.4)	37 (12.5)	48 (38.4)	82((63.6)
Prior hospitalization	128 (23.3)	–	45 (36.0)	83 (64.3)
Nursing home residence	51 (9.3)	–	7 (5.6)	44 (33.8)

*SD* Standard deviation, *COPD* Chronic obstructive pulmonary disease, *GOLD* Global initiative for chronic obstructive lung disease, *PSI* Pneumonia severity index, *ICU* Intensive care unit, *ICP* Immunocompromised group

### Antibiotic regimens and microbiology

For initial treatment in the ED, 305 (55.6%) patients were prescribed one antibiotic and 244 (44.4%) were prescribed a combination regimen. Eighty-five percent of prescriptions complied with the SEPAR guidelines (for the purpose of study, β-APS use was considered non-concordant). Empirical antimicrobial regimens are summarized in Table 2**.** The most frequently prescribed antibiotics in monotherapy were levofloxacin in 129 (42.3%) and amoxicillin-clavulanate in 108 (35.4%). Ceftriaxone plus azithromycin (in 190, 34.2%) was the most common combination. In 78 patients (14%) antipseudomonal β-lactam (β-APS) was prescribed in monotherapy or in combination. The empirical use of β-APS differed between groups and was significantly more frequent in ICP and HCAP (CAP, 6.4%; ICP, 26.4%; HCAP, 19.4%; *p* < 0.001).

**Table 2:** Description of initial antibiotic therapy for 549 inpatients

Initial therapy	Total *n* = 549	CAP *n* = 295	ICP *n* = 125	HCAP *n* = 129
**Monotherapy**	305 (55.6)	156 (52.9)	64 (51.2)	85 (65.9)
Amoxicillin-clavulanate	108 (35.4)	56 (35.9)	14 (21.9)	38 (44.7)
Levofloxacin	129 (42.3)	76 (48.7)	24 (37.5)	29 (34.1)
Ceftriaxone	22 (7.2)	13 (8.3)	7 (10.9)	2 (2.4)
**β-APS**	45 (14.8)	11 (7.1)	18 (28.1)	16 (18.8)
Piperacillin-tazobactam	26 (8.5)	3 (1.9)	12 (18.8)	11 (12.9)
Cefepime	3 (1.0)	2 (1.3)	1 (1.6)	0 (0.0)
Meropenem	16 (5.2)	6 (3.8)	5 (7.8)	5 (5.9)
Others	1 (0.3)			
**Combination**	244 (44.4)	139 (47.1)	61 (48.8)	44 (34.1)
Ceftriaxone + macrolide	190 (77.9)	119 (85.6)	41 (67.2)	30 (68.2)
β-APS + quinolone or aminoglycoside or macrolide	33 (13.5)	9 (6.5)	15 (24.6)	9 (20.5)
Others	21 (8.6)	11 (7.9)	5 (8.2)	5 (11.4)

*β-APS* Antipseudomonal β-lactam

We collected blood cultures in 340 episodes, and 145 valid respiratory specimens were retrieved. An etiological diagnosis was made in 184 patients (33.5%): 50 (40%) in the ICP group, 103 (35%) in the CAP group and 31 (24%) in the HCAP group (*p* = 0.02). *Streptococcus pneumoniae* was the most frequently isolated pathogen (124/549, 22.6%): the pneumococcal urine antigen test was positive in 111/389 (28.5%) of patients evaluated. Other microorganisms had a lower frequency: *Haemophilus influenzae* 16 (2.9%), *Moraxella catarrhalis* 11 (2%) and Enterobacteriaceae 10 (1.8%). *Pseudomonas aeruginosa* was isolated in 9/549 (1.6%): three cases in the CAP group, two in the ICP group and four in the HCAP group. Moderate-severe COPD was present in five and nine patients respectively and a previous isolate of *Pseudomonas aeruginosa* was documented in two of them. Bacteremia occurred in only one patient, with chronic dialysis.

**Fig. 1:**
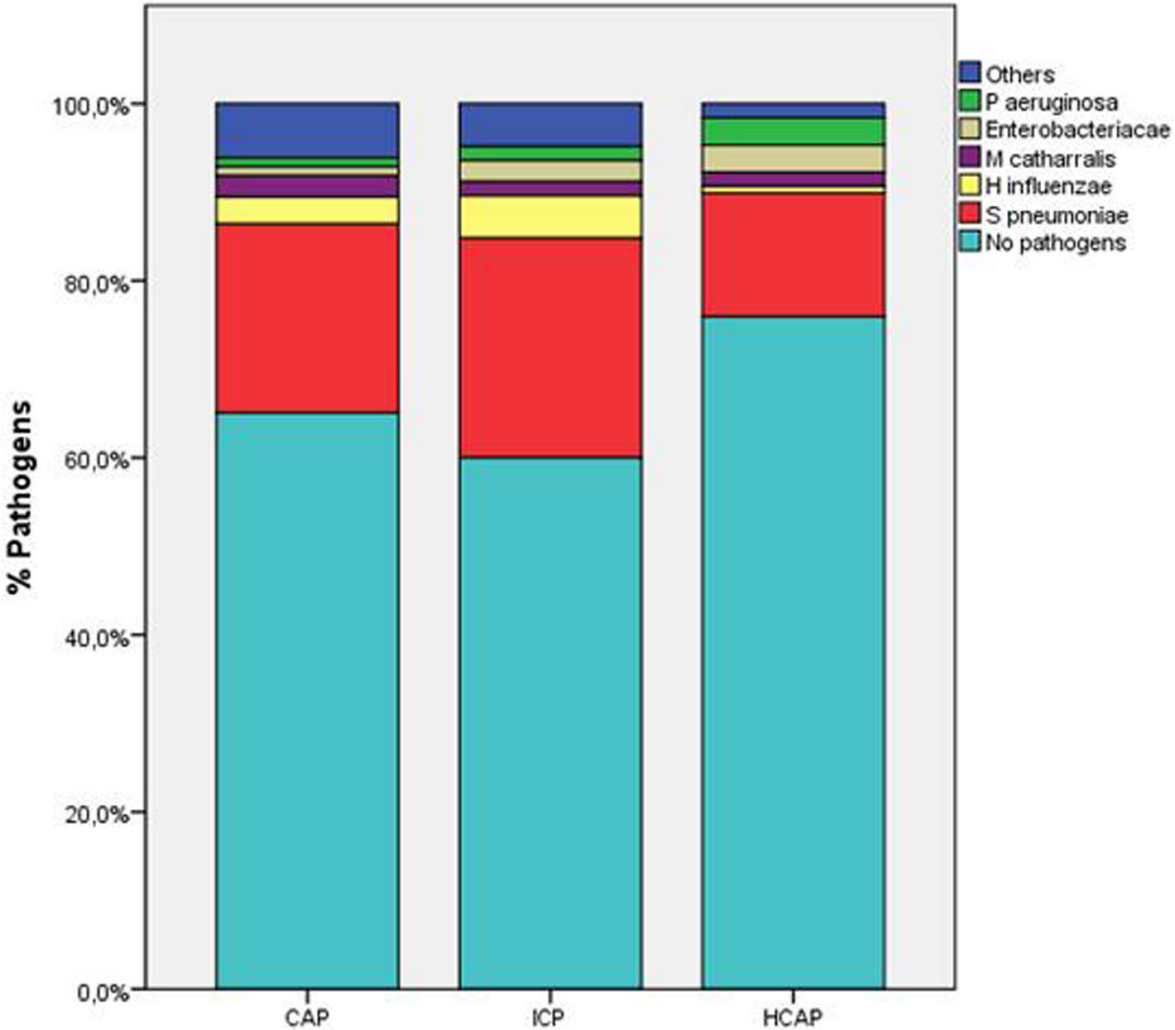
Distribution of pathogens

The distribution of microorganisms across the groups is shown in Fig. 1.

### Determinants of empirical antipseudomonal use

The main factors determining selection of antipseudomonal antibiotics are summarized in Table 3. Two patterns of utilization were documented. The first was based on monotherapy with levofloxacin, which was not administered with antipseudomonal intention and was restricted to patients with low severity-of-illness. Only three patients in the ICU received quinolones; even in non-ICU patients, these agents were mainly prescribed in patients with PSI I-III and limited radiological extent. In contrast, negative pneumococcal antigen was a key factor in the prescription of levofloxacin. The other documented pattern was based on the prescription of β-APS in monotherapy (or in combination), used for antipseudomonal coverage. Site of care was the most important determinant of β-APS prescription: in fact, β-APS was the initial treatment in 22/59 (35.6%) patients in the ICU and in 56/490 (11.4%) in ward-admitted patients (*p* < 0.0001). This association was confirmed in the multivariate analysis (OR: 8.16; 95% CI: 3.69–18.06; *p* < 0.0001).

**Table 3:** Analysis of factors associated with empirical antipseudomonal use in pneumonia. Univariate and multivariate logistic regression analysis

	Quinolones				Beta-APS			
	Univariate		Multivariate		Univariate		Multivariate	
	OR CI 95%	*p* value	OR CI 95%	*p* value	OR CI 95%	*p* value	OR CI 95%	*p* value
Male sex	0.781 (0.520- 1.173)	0.234			1.971 (1.127- 3.446)	0.017	1.316 (0.660- 2.625)	0.436
ICU admission	0.155 (0.048- 0.503)	0.002	0.256 (0.076- 0.863)	0.028	4.608 (2.538- 8.368)	< 0.001	8.166 (3.691- 18.065)	< 0.0001
PSI risk class I-III vs IV/V	0.577 (0.384- 0.866)	0.008	0.595 (0.390- 0.907)	0.016	3.440 (1.770- 6.688)	< 0.001	1.817 (0.861- 3.834)	0.117
Multilobar involvement	0.457 (0.281- 0.744)	0.002	0.531 (0.319- 0.885)	0.015	1.676 (1.021- 2.752)	0.041	1.355 (0.732- 2.507)	0.333
**Comorbidities**								
Liver disease	0.304 (0.107- 0.867)	0.026	0.413 (0.141- 1.214)	0.108	2.839 (1.412- 5.705)	0.003	1.808 (0.767- 4.261)	0.175
Bronchiectasis	0.851 (0.311 -2.326)	0.753			1.629 (0.590- 4.499)	0.346		
Diabetes	1.179 (0.771 -1.802)	0.446			0.979 (0.579 -1.655)	0.936		
Chronic renal disease	1.354 (0.798 -2.299)	0.261			0.829 (0.407 -1685)	0.604		
Chronic heart disease	1.197 (0.737 -1.943)	0.468			1.078 (0.594 -1.954)	0.806		
**COPD**								
No COPD	**Reference category**				**Reference category**			
GOLD I-III	1.061 (0.630-1.789)	0.823			0.987 (0.505-1.931)	0.971	0.790 (0.362-1.723)	0.553
GOLD IV	1.146 (0.539-2.436)	0.720			3.415 (1.653-7.055)	0.001	2.993 (1.230-7.285)	0.016
Prior hospitalization	0.836 (0.518 -1.351)	0.464			5.579 (3.370- 9.237)	< 0.001	6.760 (3.017- 15.147)	< 0.0001
Nursing home residence	0.580 (0.265-1.267)	0.172			0.488 (0.171-1.394)	0.180		
Prior antibiotic	0.897 (0.581 -1.385)	0.624			2.680 (1.645- 4.366)	< 0.001	1.058 (0.479- 2.366)	0.890
Immunocompromise	0.722 (0.439- 1.186)	0.199			3.021 (1.826- 4.999)	< 0.001	2.064 (1.135- 3.756)	0.018
Negative pneumoccocal antigen	3.768 (1.903- 7.459)	< 0.001	3.408 (1.696- 6.848)	0.001	0.754 (0.428- 1.326)	0.327		

*SD* standard deviation, *ICU* intensive care unit*, PSI* pneumonia severity index, *COPD* chronic obstructive pulmonary disease, *GOLD* global initiative for chronic obstructive lung disease

Prior hospitalization was another significant determinant of β-APS use in community-acquired pneumonia (OR: 6.76; 95% CI: 3.01–15.14; *p* < 0.0001). This factor was part of the HCAP definition and was present in up to 36% of the ICP population. In addition, an association was found between β-APS prescription and severe COPD and immunosuppression (ICP population), which are classical risk factors for *P aeruginosa* infection.

Among the 381 episodes with blood cultures and/or respiratory samples available we tested the determinants of antibiotic use as risk factors for *P aeruginosa* recovery. Only multilobar involvement (7/9 vs 119/372, *p* = 0.007), prior antibiotic (6/9 vs 109/372, *p* = 0.025) and severe COPD (3/9 vs 26/372, *p* = 0.024) presented significant associations. When prior *P aeruginosa* infection/colonization was included in the model, it was also associated with *P aeruginosa* pneumonia (2/9 vs 6/372, *p* = 0.013).

## Discussion

In this study we report the drivers of antibiotic prescription for pneumonia in the ED. Nearly 37% of hospitalized patients received antibiotics with potential antipseudomonal activity; although 14% of prescriptions were intended to provide *P aeruginosa* coverage, this pathogen was documented in only 1.6% of episodes. Severity-of-illness, identified as the need for intensive care unit admission, and prior hospitalization were the main factors in the decision to prescribe β-APS. In contrast, the prescription of levofloxacin in monotherapy was mainly associated with the presence of negative *S pneumoniae* urine antigen and less severe disease.

Few studies carried out in the ED have focused on the determinants of antibiotic use in pneumonia, even though rates of inappropriate antibiotic treatment (for whatever reason) are known to be around 50% [25]. Improving adherence to guidelines has been shown to raise the rates of appropriate treatment [26, 27]. In our study adherence to guidelines was high, around 85%, but the etiological findings show that the use of broad spectrum antibiotics could be reduced.

Therefore, although the indication for respiratory fluoroquinolones was to provide coverage against potential *S. pneumoniae* strains with reduced betalactam susceptibility, Enterobacteriaceae and particularly atypical organisms, in agreement with the SEPAR guidelines [4], the potential of levofloxacin as an antipseudomonal antibiotic should be borne in mind.

The specific determinants of the choice of levofloxacin over other options have not been studied in depth. In our study, apart from its being restricted to ward use, the most intriguing finding was its association with unilobar pneumonia, suggesting that radiological spread is interpreted as a sign of severity, as reported elsewhere [28, 29]. Another interesting finding was the association with negative pneumococcal urine antigen tests. The presence of a positive pneumococcal urine antigen test is indicative of *S pneumoniae* etiology, but therapy is based on the result in fewer than 15% of cases [30]. Although outcomes with fluoroquinolones and β-lactams seem to be similar, it is less clear whether a β lactam/macrolide combination is superior to fluoroquinolone monotherapy; the findings are conflicting and the data in patients with severe disease are insufficient to draw conclusions [31]. Thus, attending physicians should be aware of this susceptibility pattern and should bear in mind the recent EMA warning to restrict indications of fluoroquinolones due to potentially severe adverse events, due to the increased risk of cardiac events and aneurysmal rupture in the elderly; indeed, safer treatments such as β-lactams are recommended [31].

In the case of the coverage of *P aeruginosa,* the clinical practice guidelines range substantially, from a non-specific recommendation [3] to a recommendation in critical patients with specific risk factors [1, 4]. In general, and in keeping with our results, overtreatment has been reported in several studies worldwide, reaching nearly 40% of patients in some cases [11, 32–34]. However, when evaluating severe episodes, the failure to cover *P aeruginosa* is associated with increased mortality. For instance, in a multicentre study of 529 severely ill CAP admitted to the ICU, despite correct treatment according to IDSA guidelines in 15 (75%) of 20 cases of *Pseudomonas aeruginosa* infection, antimicrobial treatment at admission was inadequate. Chronic obstructive pulmonary disease, malignancy, previous antibiotic exposure, and radiographic findings demonstrating rapid spread of disease were associated with *P aeruginosa* pneumonia [9].

Few studies have evaluated aetiology according to severity-of-illness at ED presentation; Cilloniz and colleagues [35] identified Gram-negative enteric bacteria and *P aeruginosa* as being more frequent in higher-risk groups than in low-risk groups, strengthening the argument that coverage of Gram-negative organisms is needed for these patients. However, in their study, only 5% of patients admitted to a respiratory ICU presented *P aeruginosa.*

In our study, antipseudomonal coverage was provided in 22/59 of cases admitted to ICU, but in fact none of these patients were infected by *P aeruginosa*.

In severe episodes of CAP requiring vasopressors or mechanical ventilation, beta-lactam/macrolide combinations were preferred in view of the evidence of reduced mortality due to the immunomodulatory effects of macrolides [36, 37]. Moreover, in agreement with recent recommendations [38] antimicrobial de-escalation is a common practice in the ICU, once the identity of organism has been confirmed by laboratory tests.

In our study, site of care was a more decisive factor than high PSI; 35.6% of ICU patients received β-APS compared with 11.4% of ward-admitted patients (*p* < 0.0001).

Another key determinant of prescription in our study was prior hospitalization. This variable is inherent to the HCAP definition [22] in an attempt to identify patients at risk of infection by multidrug-resistant microorganisms (MDR), but it has been associated with an unjustified increase in antipseudomonal prescription in patients with pneumonia, despite the lack of any increase in nosocomial pathogens [39]. Nevertheless, several recent scores attempting to refine risk factors of MDR systematically include this factor [20, 40]. In a study performed with 935 hospitalized patients which included immunosuppressed patients, prior hospitalization was the main risk factor for MDR [20]. Although we used the traditional definition with a cut-off of 48 h, longer hospital stay has been associated with an increased risk of MDR infection [41], a factor that was not analysed in our study.

Other factors related to the use of β-APS were immunosuppression and severity of COPD, which are well-known risk factors for *P aeruginosa* infection. In our study severe COPD patients accounted for 60% of those infected; in the worldwide study by Restrepo et al (42) COPD was the main comorbidity, but immunosuppression was present in only two patients.

Determinants of *P aeruginosa* coverage used in the ED were not useful in predicting *P aeruginosa* infection in our study. However, prior *P aeruginosa* infection/colonization together with severe COPD seem to be good predictors, although multivariate analysis was not carried out due to the low number of *P aeruginosa* episodes. These results are in agreement with Restrepo et al [42] and with the 2019 ATS/IDSA guidelines for community-acquired pneumonia [43] recommendations.

The most consistent risk factor for PA is prior colonization. Unfortunately, this information is not always available at the ED of a referral center because not all CAP patients have prior isolates. In any case, our data suggest that potential prior PA respiratory colonization reported in computerized clinical records should be checked in the ED.

The main limitation of this study is its single-centre retrospective design. Furthermore, the generalizability of the findings is limited as this is a single-centre cohort from 2010 to 2011 that may no longer reflect current clinical practices or pathogen distribution; for example, moxifloxacin is not approved for systemic use in Spain, a circumstance that may limit the generalization of our findings. However, the single-centre design allowed us to avoid confounding factors such as differences in protocols or bias in patient selection.

## Conclusions

Antipseudomonal prescription was common, in spite of the very low incidence of *Pseudomonas aeruginosa*. Levofloxacin was prescribed to patients with low severity and negative pneumococcal urine antigen tests. Antibiotic prescription intended to provide *P aeruginosa* coverage was guided mainly by severity-of-illness, prior hospitalization and, less frequently, by specific risk factors. However, these factors were not associated with *P aeruginosa* episodes. Only prior *P aeruginosa* infection/colonization and severe COPD seem to be reliable in clinical practice.

## Data Availability

The datasets used in this study are available from the corresponding author upon reasonable request.

## Abbreviations

ATS: American Thoracic Society
CAP: Community-acquired pneumonia
HCAP: Healthcare-associated pneumonia
ICP: Immunocompromise
IDSA: Infectious Diseases Society of America
COPD: Chronic obstructive pulmonary disease
GOLD: Global initiative for chronic obstructive disease
PSI: Pneumonia severity index
EMA: European Medicines Agency
